# Eicosanoid Metabolomic Profile of Remdesivir Treatment in Rat Plasma by High-Performance Liquid Chromatography Mass Spectrometry

**DOI:** 10.3389/fphar.2021.747450

**Published:** 2021-09-29

**Authors:** Ping Du, Guo-yong Wang, Rui Zhao, Zhuo-ling An, Li-hong Liu

**Affiliations:** Department of Pharmacy, Beijing Chao-Yang Hospital, Capital Medical University, Beijing, China

**Keywords:** eicosanoids, remdesivir, metabolomics, COVID-19, HPLC-MS/MS

## Abstract

Remdesivir, a nucleotide analog prodrug, has displayed pharmacological activity against SARS-CoV-2. Recently, eicosanoids are widely involved in regulating immunity and inflammation for COVID-19 patients. Rats were intravenously administered remdesivir at a dose of 5 mg/kg, and series of blood samples were collected before and after treatment. Targeted metabolomics regarding the eicosanoid profile were investigated and quantitated simultaneously using the previously reported reliable HPLC-MS/MS method. Additionally, interplay relationship between metabolomics and pharmacokinetic parameters was performed using the Pearson correlation analysis and PLS model. For the longitudinal metabolomics of remdesivir, metabolic profiles of the same rat were comparatively substantial at discrete sampling points. The metabolic fingerprints generated by individual discrepancy of rats were larger than metabolic disturbance caused by remdesivir. As for the transversal metabolomics, the prominent metabolic profile variation was observed between the baseline and treatment status. Except for TXB2, the inflammatory- and immunology-related eicosanoids of resolvin D2, 5-HEPE, 5-HETE, and DHA were significantly disturbed and reduced after single administration of remdesivir (*p* < 0.05, *p* < 0.001). Moreover, the metabolite of PGE2 correlated with GS-441524 (active metabolite of remdesivir) concentration and pharmacokinetic parameters of C_max_, AUC_0-t_, AUC_0-infinity_, and CL significantly. Eicosanoid metabolic profiles of remdesivir at both longitudinal and transversal levels were first revealed using the robust HPLC-MS/MS method. This initial observational eicosanoid metabolomics may lighten the therapy for fighting COVID-19 and further provide mechanistic insights of SARS-CoV-2 virus infection.

## Introduction

Since December 2019, coronavirus disease 2019 (COVID-19) has strained the global healthcare system seriously (Zhu et al., 2020). In the face of the current global pandemic posed by severe respiratory syndrome coronavirus 2 (SARS-CoV-2) infection, there is an urgent necessitation not only to prompt a fervent search for effective therapy but also to improve our knowledge of the metabolomic mechanism of this disease. Nowadays, several approaches are being investigated, including small molecular chemical antiviral drug (Costanzo et al., 2020), traditional Chinese medicine (Ren et al., 2020), and vaccines (Lurie et al., 2020), which has improved the ratio of benefit/risk of patients with COVID-19 significantly.

A nucleotide analog prodrug, remdesivir, has broad-spectrum activity against a variety of viruses, such as Ebola, SARS-CoV-2, Middle East respiratory syndrome coronavirus (MERS-CoV), and COVID-19 *in vitro* and *in vivo* (Lamb, 2020). Nowadays, remdesivir was authorized by the United States Food and Drug Administration for emergency use in November 2020 for treating patients with severe COVID-19. Undoubtedly, clinical treatment of remdesivir has brought obvious benefit for patients with COVID-19 to some extent.

Metabolomics is one of the most powerful tools for studying the interaction between genetic background and exogenous and endogenous factors in human health. Pharmacometabolomics can provide a metabolic profile or metabotype variation induced by drug treatment, which straightly mirrors the metabolic status of small molecules between tissues and fluids and facilitates better understanding of biological processes related with the disease. Endogenous small-molecule metabolites are indispensable for vital infection, which can provide fundamental material for rapidly proliferating and constructing nucleic acid, proteins, and membrane (Thomas et al., 2020). As one of the key components for physiological function, eicosanoids not only play a crucial role in the pathological process of allergy, inflammation, and cancer (Harizi et al., 2008; Wang and DuBois, 2010; Dennis and Norris, 2015) but also promote the cytokine storm of SARS-CoV-2 infection (Hammock et al., 2020). It is now well known that general alterations in metabolomic profiles and trajectories, which are a link between genotypes and phenotypes and provide terminal information, can provide crucial comprehension of SARS-CoV-2 infection pathogenesis (Thomas et al., 2020). Unfortunately, the metabolic mechanism of remdesivir was not fully figured out, especially with regard to eicosanoid metabolite reprogramming/perturbation.

Limited literatures have reported the metabolite changes among COVID-19 patient cohorts, such as cytosine and tryptophan–nicotinamide pathways (Blasco et al., 2020), lipids (Archambault et al., 2021), amino acid, and fatty acid (Shen et al., 2020). However, as far as we know, no eicosanoid metabolic profiling literatures were reported pertaining to remdesivir treatment both *in vitro* and *in vivo*. It is therefore reasonable and feasible to study the association of eicosanoid metabolic profiles and remdesivir treatment. In view of the shortcomings described above, the purpose of the present study was originally proposed to longitudinally and transversally investigate the eicosanoid metabolic fingerprint induced by remdesivir treatment in rats. Furthermore, correlation analysis was investigated between eicosanoids metabolomic and pharmacokinetics. Overall, we provide the first deep interrogation of eicosanoid changes that benefit propitious understanding of how remdesivir interacted with small molecular metabolites. The results of the present study will shed light on how remdesivir combats SARS-CoV-2 virus from the perspective of metabolomic perturbation.

## Material and Methods

### Chemicals

Both the unlabeled chemical standards and stable isotope labeled internal standards (IS) were obtained from Cayman Chemical. Detailed chemicals were shown in our previous literature (Du et al., 2020). Remdesivir was purchased from DC Chemicals Company. The purity of these standards was ≥98%. HPLC-grade methanol (MeOH), acetonitrile (ACN), and isopropanol (IPA) were utilized for preparing stock or working solutions and mobile phases. Ultrapure water was produced using the Milli-Q reference water purification system.

### Experimental Animals

Animal experiments were carried out in accordance with the guidelines for the care and use of laboratory animals (published by the National Institutes of Health, NIH publication number 85–23, revised in 1996). Six (eight-week-old, body weight of 180–220 g) male Sprague–Dawley rats were obtained from Beijing Vital River Laboratory Animal Technology Co., Ltd. (Beijing, China). Rats were kept in temperature (25 ± 2 °C)/humanity (40–70%)-controlled unidirectional airflow room, and 12 h light on/off cycle was provided.

### HPLC-MS/MS System

LC-20ADXR system (Shimadzu, Japan) coupled with QTRAP 5500 spectrometry (SCIEX, Canada) was adopted. Metabolomics was performed in accordance with the previously reported method (Du et al., 2020). The chromatographic column was a Waters HPLC BEH C_18_ (2.1 × 100 mm, 1.7 μm, Milford, United States) set at 55°C. Detailed parameters of liquid chromatography and mass spectrometry are all shown in Supplementary Table S1 and our previous literature (Du et al., 2020).

### Metabolomic Profiling of Remdesivir Treatment

For the longitudinal pharmacometabolomics of remdesivir, animals were accommodated for 1-week prior experiment under controlled environment. Rats were intravenously administered remdesivir dissolved with 12% sulfobutylether-β-cyclodextrin in water at a dose of 5 mg/kg. Blood samples (approximate 500 μl) were collected from ophthalmic veins by sterile capillary into (NaF/K-Ox) tubes before (0 h) and after administration (5, 15, 30 min, 1, 2, 4, 8, 12, 24, and 48 h), and then directly centrifuged to obtain plasma (3,500 rpm, 10 min, 4°C). All plasma samples were kept at −80°C for further analysis.

For the transversal pharmacometabolomics of remdesivir, raw metabolomic data were separated into two parts: before (pre-dose) and after (post-dose) intravenous administration. The metabolomic profiling and trajectory effect of remdesivir were compared and analyzed using various statistical analysis methods.

### Sample Preparation

All internal standard (IS) solution was proposed to obtain concentration of 10 ng/ml. Mixed standard solution was prepared as listed in Supplementary Table S1 and diluted gradually using methanol to establish the calculation curve (ranged of 0.005–500 ng/nl). Hence, an aliquot of 20 μl standard mixture was spiked with 10 μl IS mixture, 10 μl water, and 40 μl methanol to prepare the calibration curve samples in succession.

As for the unknown plasma samples, the protein precipitation method was utilized to prepare injection samples as previously reported in our study (Du et al., 2020). Briefly speaking, an aliquot of 20 μl plasma was spiked with 10 μl of IS mixture solution and 50 μl methanol to prepare analytical samples. The resulting mixture was then vortexed for approximate 1 min. After centrifugation at 13, 500 rpm for 10 min at 4°C, the supernatant was collected and infused into the HPLC-MS/MS system.

### Pharmacokinetic Analysis of Remdesivir

It is well known that GS-441524 (Nuc) is the main active metabolite of remdesivir. In view of the pivotal role for therapy during the COVID-19 pandemic, a selective, robust, and rapid HPLC-MS/MS method was also developed and fully validated in our previous study (Du et al., 2021). The detailed validation parameters are listed in Supplementary Table S2. Chromatography separation was accomplished on Waters XBrige C_18_ column (50 × 2.1 mm, 3.5 μm) using gradient elution. The calibration curve was linear in the range of 2–1,000 ng/ml (Nuc). One-step protein precipitation was used for plasma preparation. Pharmacokinetic parameters including C_max_ (maximum concentration), AUC (area under the curve), MRT (mean residence time), and CL (clearance) were calculated using Phoenix WinNonLin (Pharsight 8.3, Mountain View, CA) software.

### Correlation Analysis Between Metabolomics and Pharmacokinetics

In order to investigate the interplay between metabolomics and drug exposure, correlation analysis was made using the Pearson correlation coefficient. Next, the concentration of Nuc (metabolite of remdesivir) and different metabolite intensities were also correlated. Only connections with a *p* value of < 0.05 and r ≥ 0.5 were retained, and *p* < 0.05 was set for statistical significance (Hu et al., 2020). A supervised partial least squares (PLS) model was employed for correlation analysis between metabolic data and pharmacokinetic parameters (e.g., AUC and C_max_).

### Quality Assurance for the Analysis

First, for the purpose of ensuring reliable quantitation of all analytes and better comparability in routine analysis, quality control (QC) samples were performed by pooling equal volumes of unknown plasma from all the unknown plasma samples. QC control chart has been employed to assure the data quality. Hence, six aliquots of pooled QCs were prepared and the analytical sequence was interpolated to check the status of the HPLC-MS/MS system.

Second, apart from the prerequisite approach using simulated plasma samples during the analytical batch, the additional solution QC samples were also implemented to obtain reliable results. The calibrations and QCs should be investigated under the acceptance criteria issued by bioanalytical method validation guidance (Evaluation and Research, 2018).

Third, uncertainty of measurement, which consists of type A and type B uncertainty, was simultaneously adopted not only to assure the analytical reproducibility but also to verify if the material analyzed falls in the scope of method validation. The QC solution at concentration of 1 ng/ml (*n* = 15 with three batches) was prepared and investigated from the aspect of reliable quantitation.

### Data Processing and Statistical Analysis

Raw data files were processed and checked by MultiQuant 3.0.1 (SCIEX). The concentrations of analytes were calculated according to the calibration curve. Metabolite values were median-scaled and log-transformed to normalize metabolite distributions, and the unit variance (UV) scale was employed for all datasets. SIMCA-P software 14.1 (Umetrics AB, Umeå, Sweden) was utilized for principal component analysis (PCA), orthogonal partial least squares discriminant analysis (OPLS-DA), and PLS. Metabolites of interest were picked up according to the values of variable importance in the projections (VIP>1). Metaboanalyst 5.0 (https://www.metaboanalyst.ca/) was also utilized for hierarchical cluster analysis (HCA) and t-test. Quantitative data were analyzed by IBM SPSS 26.0 (Armonk, New York, United States). A *p* value of ≤0.05 was considered statistically significant.

## Results

### Overview of Eicosanoid Metabolomics for Remdesivir

**FIGURE 1:**
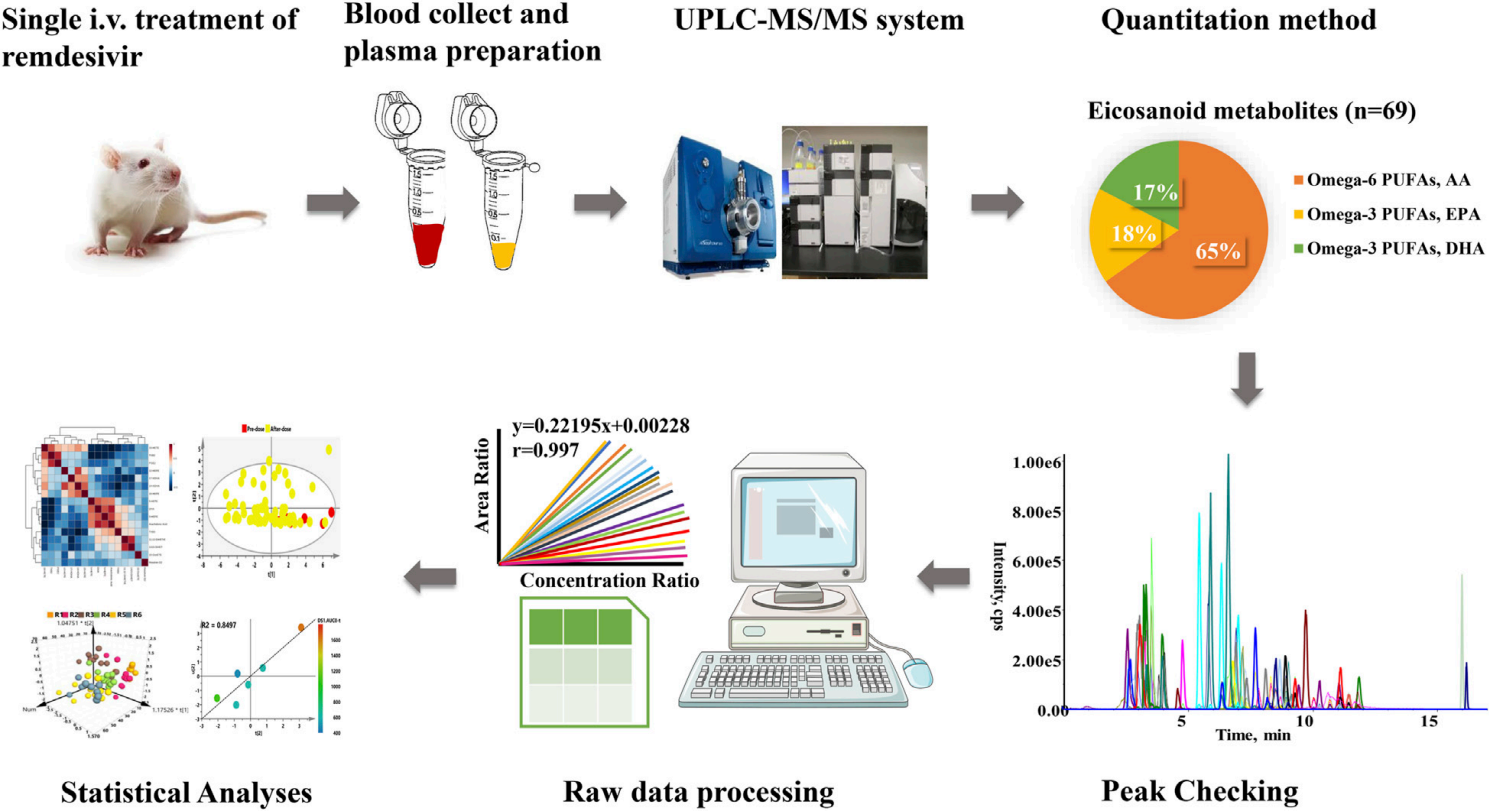
Workflow of eicosanoids metabolomics applied in this study.

The eicosanoid metabolomic method was utilized for present quantitation developed in our laboratory (Du et al., 2020). As shown in Figure 1, 69 eicosanoid metabolites, which contained omega-3– and omega-6–derived polyunsaturated fatty acid (PUFA), were included in present study. All biologically active metabolites can be quantitated after a single injection. The calculation linearity ranged 0.005–500 ng/ml with lower limit of quantification (LLOQ) of 5 pg/ml, which provides powerful capability for successful quantitation of low abundance compounds. Furthermore, other method parameters were all carefully investigated (Supplementary Table S1).

### Longitudinal Metabolic Profiling Analysis of Remdesivir

**FIGURE 2:**
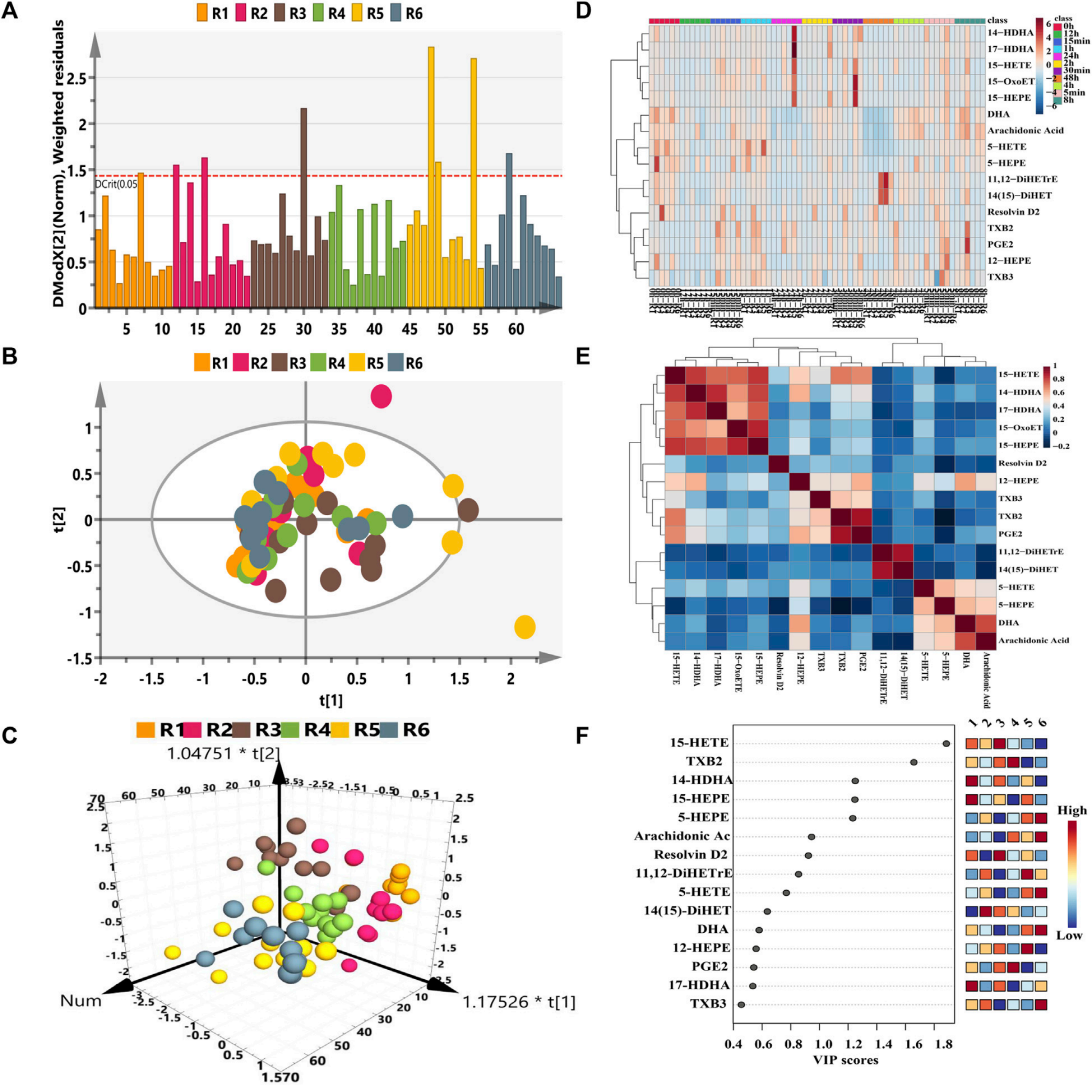
Longitudinal eicosanoid metabonomic fingerprints of remdesivir. **(A)** DModX analysis for data quality. **(B)** PCA score plots from six rats. **(C)** OPLS-DA score plots from six rats. **(D)** Hierarchical cluster heatmap of all metabolites at different time points. The color scales mark relative intensity after normalization and scaling. **(E)** Differential metabolite correlation heatmaps for plasma samples. The color scale **(right)** indicates the degree of correlation of metabolites (red-positive correlation, blue-negative correlation). **(F)** Metabolites of VIP values > 1.

With respect to the longitudinal metabolic fingerprints of remdesivir, metabolite peak area ratios of all plasma samples from six rats were pooled into statistical dataset. The distance to model (DModX) plot was employed to check the outliers. As described in Figure 2A, only three samples exceed the limit of 2. PCA and OPLS-DA analysis were employed to co-analyze all observations to explore the longitudinal metabolic trajectory of all rats. From the results of Figures 2B,C, plasma samples of individual rat were almost divided into tight clusters, which indicated that the longitudinal metabolic characteristic of individual rat was relatively stable after remdesivir treatment. The metabolic fingerprint changes generated by individuals were larger than the metabolic disturbance induced by remdesivir. The results of Figure 2D indicated each sampling appeared with a special metabolic profile. Differential metabolite correlation heatmaps for plasma samples are illustrated in Figure 2E. Among the eicosanoid cascade metabolites, the metabolites correlated with each other positively or negatively. The high negative correlation was displayed for most of the metabolites. As described in Figure 2F, the VIP of each metabolite was analyzed according to the established OPLS-DA model. The number of VIP > 1 was 5 for all datasets.

### Transversal Metabolomics of Remdesivir

**FIGURE 3:**
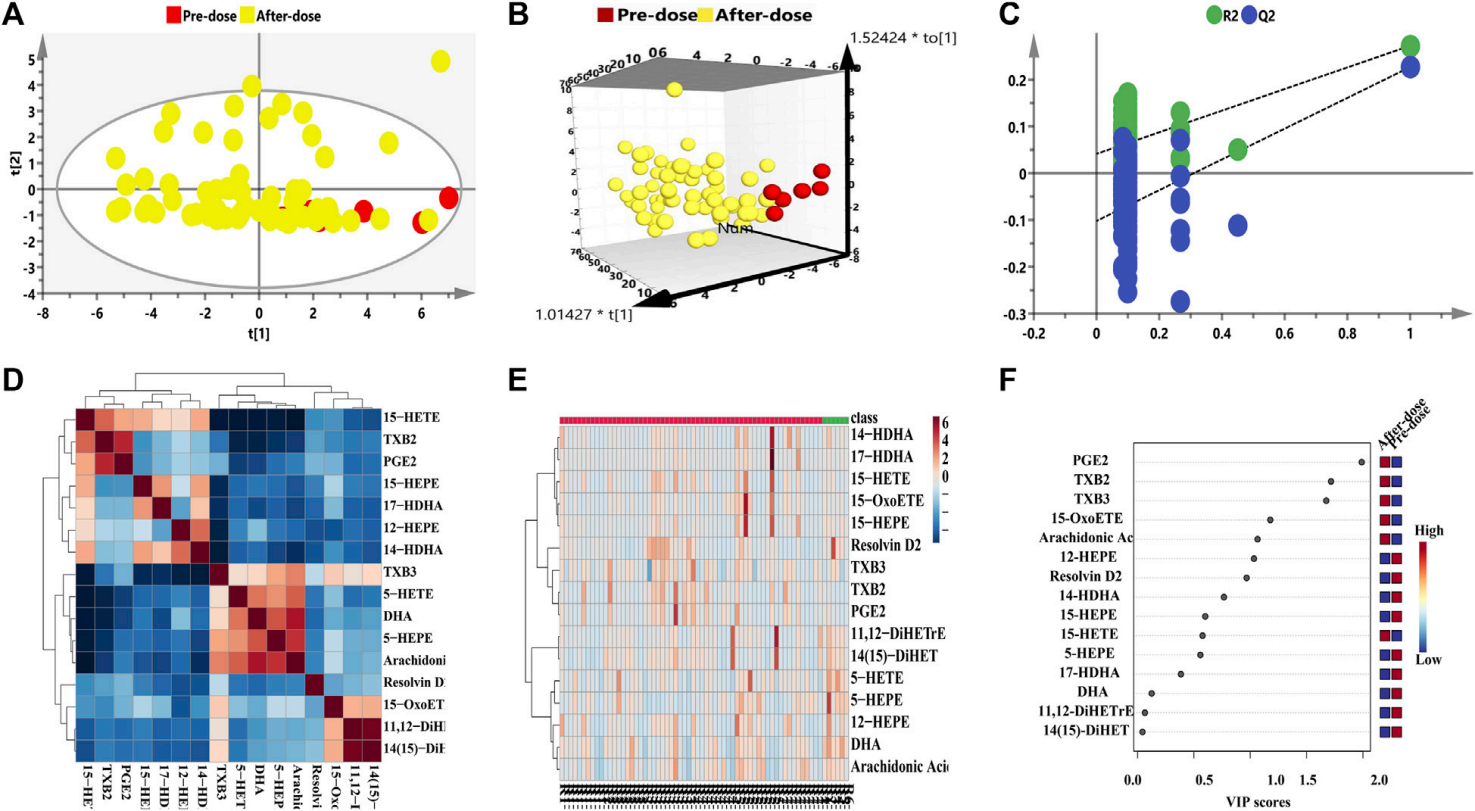
Eicosanoid metabolic profiling of remdesivir before and after treatment. **(A)** PCA score plot; **(B)** OPLS-DA score plot; **(C)** Random permutation test with 100 iterations, and no overfitting was observed. **(D)** Hierarchical cluster analysis of metabolite–metabolite correlation in response of remdesivir treatment. **(E)** Heatmap clustering of eicosanoid metabolomic profiling before and after treatment by remdesivir. **(F)** Rank of the different metabolites (the top 10) according to the VIP score and the coefficient score.

The metabolomic features of plasma samples at baseline (pre-dose) were compared with those at the treated period (post-dose) in order to examine the metabolic phenotype variation caused by remdesivir. For the transversal pharmacometabolomics of remdesivir, Figures 3A,B illustrated all metabolic data from six rats were introduced for unsupervised PCA and OPLS-DA analysis. Although limited metabolic samples were used in the present study at baseline time points, the individuals of both groups were discriminated well in the OPLS-DA model. Moreover, the random permutation test with 100 iterations was employed to investigate validity and predictability of the OPLS-DA model. Figure 3C showed no overfitting was observed for all introduced data [R2 = (0.0, 0.0413), Q2 = (0.0, -0.102)]. In addition, hierarchical cluster analysis was also carried out to investigate transversal metabolic disturbance. As shown in Figure 3D, the metabolites correlated with each other positively (e.g., PGE2, TXB2, 5-HEPE, and DHA) or negatively (e.g., TXB3, 14-HDHA, 15-HEPE, and arachidonic acid). The individual metabolic data are shown in Figure 3E, which indicated that there was a visible discrimination between pre- and post-dosage. The clustering heatmap provided an overview of all eicosanoid in plasma before and after intravenous administration, indicating the fluctuant levels of relative increase (brown) and decrease (blue). To find potential significance of metabolites associated with the treatment of remdesivir, the VIPs were determined in view of the established OPLS-DA model. The VIP values larger than 1 were set the most significant influence, with a plot shown in Figure 3F. A total of seven metabolites were screened out with VIP > 1. Taken together, the above data indicated that remdesivir can perturbate the eicosanoid metabolic profile *via* cyclooxygenase, cytochrome 450, and lipoxygenase pathways. Inherent metabolic phenotype variations had taken place as a result of the treatment of remdesivir.

**FIGURE 4:**
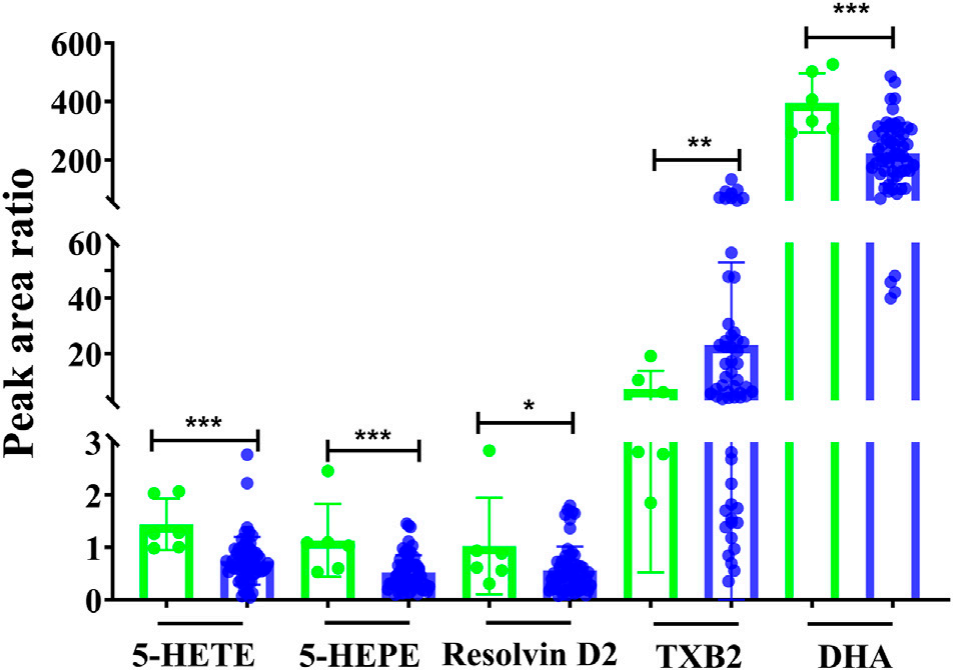
Metabolic peak area ratio comparation between the pre-dose and post-dose. *p < 0.05, ***p < 0.001, and two-tailed unpaired t-test.

Additionally, the metabolic intensity was also compared between pre-dose and post-dose. As shown in Figure 4, the inflammatory- and immunology-related eicosanoids of resolvin D2, 5-HEPE, 5-HETE, and DHA were significantly disturbed and reduced after single administration of remdesivir (*p* < 0.05, *p* < 0.001). On the other hand, TXB2 was significantly increased compared with the pre-dose status (*p* < 0.001).

### Correlation Study Between Specific Metabolite and Pharmacokinetic Parameters

After intravenous administration, remdesivir is rapidly converted into the active metabolites of Nuc in blood. The t_max_ of Nuc ranged 0.5–1 h with a median of 0.5 h.

**FIGURE 5:**
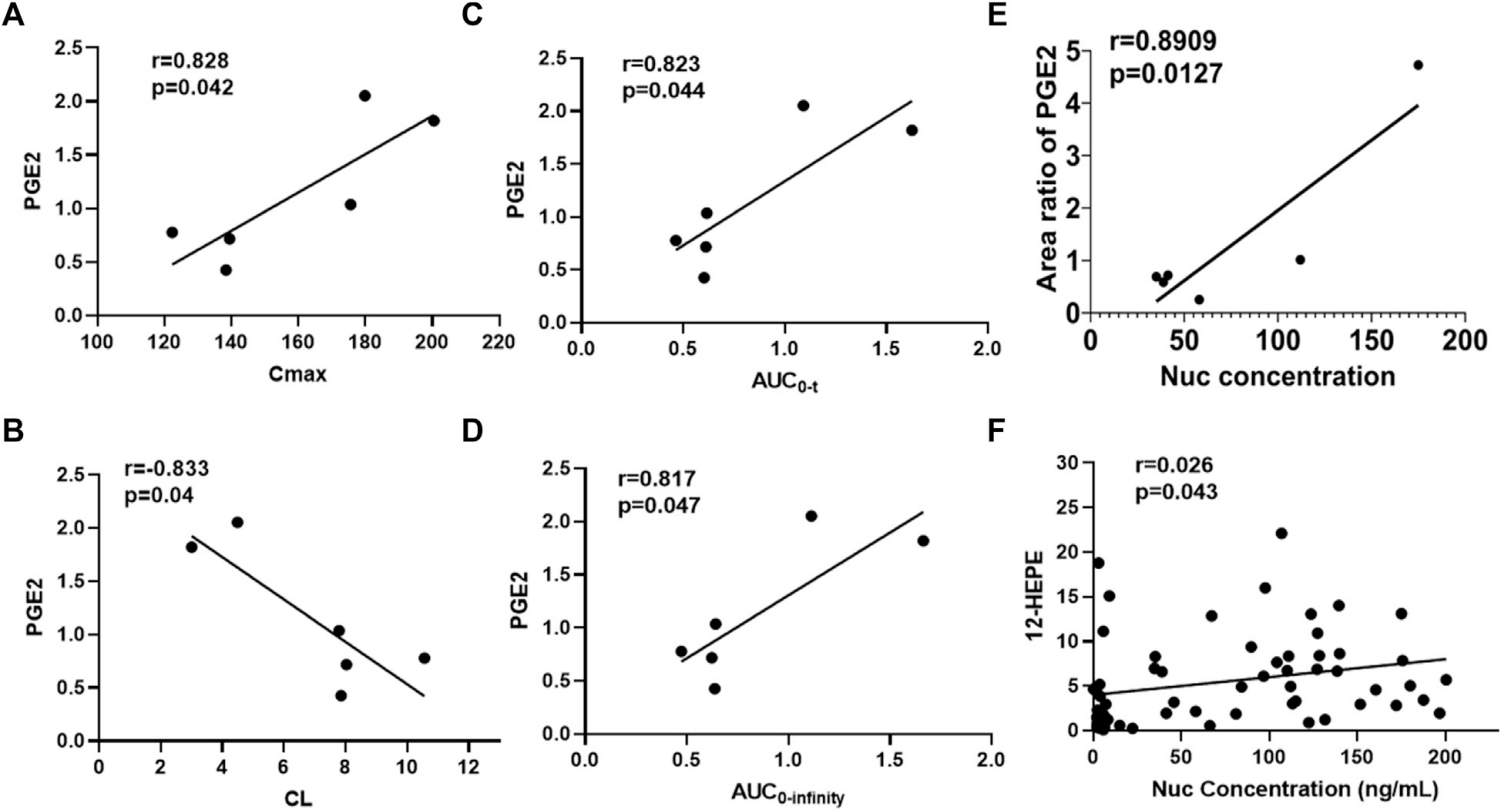
Correlation analysis between metabolites and pharmacokinetic parameters of Nuc. **(A)** Area ratio of PGE2 *vs.* C_max_; **(B)** Area ratio of PGE2 *vs.* CL; **(C)** Area ratio of PGE2 *vs.* AUC_0-t_; **(D)** Area ratio of PGE2 *vs.* AUC_0-infinity_; **(E)** Area ratio of PGE2 *vs.* Nuc concentration (4 h after administration of remdesivir); **(F)** Area ratio of 12-HEPE *vs.* Nuc plasma concentrations.

To further explore whether metabolic fingerprint disturbance accompanies the plasma drug exposure, the Pearson correlation analysis was conducted to determine what metabolites highly interplay with this tendency. As shown in Figure 5 **,** the metabolite of PGE2 correlated with C_max_, AUC_0-t_, AUC_0-infinity_, CL, and Nuc concentrations significantly (correlation coefficient (r)>0.8, *p* < 0.05). Figure 5F highlighted the interrelated relationship between 12-HEPE and all concentrations of Nuc despite the lower correlation coefficient. Overall, the Pearson correlation analysis displayed a strong relationship between specific metabolic alterations and pharmacokinetic parameters.

It is reported that AUC and C_max_ can be regarded as indicators of drug efficacy or toxicity to some extent (Xing et al., 2019). Therefore, AUC and C_max_ were chosen for further PLS model analysis. A supervised PLS model was constructed for its capability to predict PK parameters and identify the relationship between two sets of variables (Xing et al., 2019). Thus, the 16 metabolites were described as one set of variables (*X*, the predictive variables), while AUC or C_max_ was represented as set of variables (*Y*, the response variables) (An et al., 2021). First, the PCA model was constructed to examine outliers to avoid deviation of prediction. All rats were scattered in the PCA score plot according to the distance to the model plot (Supplementary Figure S1).

**FIGURE 6:**
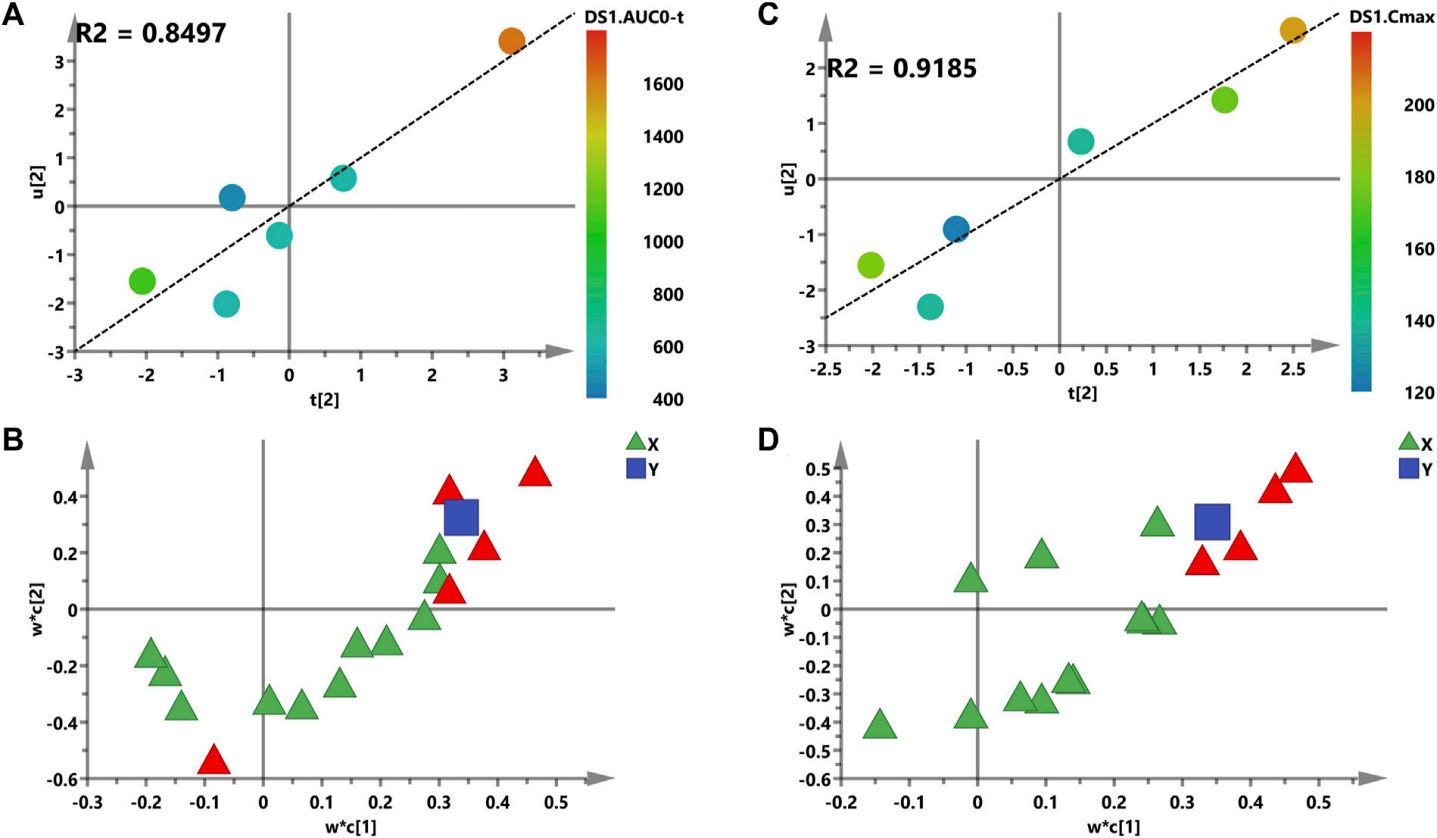
PLS models of pre-dose metabolic characteristics for predicting PK parameters of Nuc. **(A,C)** Score plots for the variables of AUC and the C_max_ prediction model; a color from blue to red represents the response variable from low to high. **(B,D)** Loading plots for AUC and the C_max_ prediction model. The blue box represents the response variable; each triangle represents a metabolite, and the triangles in red represent the metabolites with VIP >1.0.

Second, the intensities of 16 metabolites were matched to AUC or C_max_ of Nuc in the PLS model in order to probably assess the relevance between the *X* and *Y* variables (Figure 6). The two-component PLS model is adopted for AUC and C_max_ prediction, which indicates a visible positive linear regression (Figure 6A, R^2^ = 0.8497; Figure 6C, R^2^ = 0.9185). Figures 6B,D shows the loading plot of the above models, and the relevance between *X* (triangle) and *Y* (box). As shown in this loading plot, *X* variables on the top right or low left corner represent positively or negatively correlated to AUC or C_max_, respectively. Besides, five (AUC) and four (C_max_) VIP > 1.0 *X* variables were identified due to the contribution of *X* variables to the PLS model (red triangles, Figures 6B,D), and PGE2 was the most modified metabolite with VIP > 1, *p* < 0.05 (data not shown).

### Quality Assurance and Uncertainty of Measurement

**FIGURE 7:**
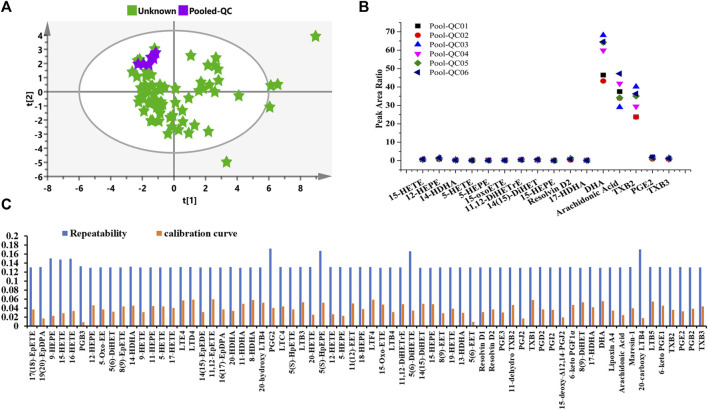
Quality control performance and uncertainty measurement for the eicosanoid metabolic quantitation. **(A)** Unsupervised PCA score of pooled QC plasma samples. **(B)** Control chart of pooled QCs in the present study. **(C)** Uncertainty of measurement of solution QC sample at a concentration of 1 ng/ml regarding quantitative repeatability and calibration curve.

For the sake of obtaining reliable and reproducible results, several approaches and all sources of fluctuations have been identified and taken to minimize unwanted variation/bias, such as sample handling and preparation, and HPLC-MS/MS system status. As shown in Figure 7A, the peak area ratios of pooled QC plasma samples were clustered under unsupervised PCA analysis. Figure 7B indicated that the fluctuation of pooled QC samples was constantly pertaining to each analyte, which represented that the evenly interspersed pooled QCs were efficacious and robust throughout the analytical procedure.

The “bottom-up” approach was utilized to correctly determine measurement uncertainty (UM). Sources of uncertainty and cause-and-effect diagram during the operating procedure were identified and estimated according to our previously reported literature (Du et al., 2019). In addition to the same operation of stock solution, sample preparation, extraction recovery, HPLC-MS/MS error, the calibration curve, and repeatability were examined using QC solution samples at the concentration of 1 ng/ml. Besides, as descripted in Figure 7C, the uncertainties of measurement were almost constant under present quantitating conditions. The largest uncertainties for repeatability and calibration curves were no more than 0.18 for all analytes. Overall, both the fluctuating control chart and uncertainty of the measurement diagram demonstrate the metabolomic results can be further utilized for statistical analysis.

## Discussion

Since the first detection in December 2019, the COVID-19 pandemic has wreaked havoc worldwide. To the best of our knowledge, no investigation is currently available regarding the metabolic perturbations and the relationship between eicosanoid metabolic fingerprint and remdesivir treatment. The main purposes of this research were to explore the eicosanoid metabolic profiling after treatment by remdesivir in rats for better understanding the pathogenesis of COVID-19 and further comprehending the mechanism of remdesivir.

One of the observed common symptoms of COVID-19 is systemic inflammatory response in the lung (e.g., pneumonia, fever, and multi-organ failure) caused by SARS-CoV-2 infection (Azkur et al., 2020; Mehta et al., 2020). Additionally, coronavirus infection led to tissue damage and subsequently triggers endoplasmic reticulum stress response as well as eicosanoid cytokine storms. Eicosanoids stimulate the resolution of inflammation and alleviate systemic hyperinflammatory responses by modulating the endoplasmic reticulum stress response (Mehta et al., 2020). Infectious processes often activate the formation of inflammasomes, which subsequently form an eicosanoid storm composed of pro- and anti-inflammatory mediators, thereby disrupting the time course and resolution of inflammation (Dennis and Norris, 2015). Thus, exploring the eicosanoid metabolic characteristics and mechanism has become a high priority in determining management strategies to block the spread of SARS-CoV-2, and targeting eicosanoids may provide a new therapeutic approach to combat COVID-19.

It is noteworthy that metabolomics can not only provide a nearly instantaneous metabolite measurement but also map specific metabolome during a normal or abnormal physiological condition, which renders metabolomics a powerful means to assess response to drug, disease states, and metabolic effects mediated by infection and immunology (Nicholson et al., 2012; Diray-Arce et al., 2020; Du et al., 2020). Eicosanoids (e.g., prostaglandins, thromboxanes, and leukotriene), which are derived from oxygenated polyunsaturated fatty acids (PUFAs), have been regarded primarily as a pro-inflammatory mediator of inflammation, immunity, and allergy (Hammock et al., 2020). Figure 1 presented the workflow of determination; a volume of 20 μl plasma was utilized for analysis. To fully characterize remdesivir-induced perturbations of eicosanoid metabolites, both the omega-3 and omega-6 sources of metabolites were quantified. After careful chromatography peak double-checking by different experimenters, the raw data were imported to software for further statistical or descriptive analyses. Furthermore, both the longitudinal and transversal metabolomics of remdesivir were evaluated to reveal the metabolic trajectories, and reliable quality assurances through the present study guarantee high-quality data.

In our study, the eicosanoids cascade metabolites assembled together with each rat (Figures 2B,C) after single administration of remdesivir. The most obviously disturbed metabolites shown in Figure 2F included the lipoxygenase pathway (15-HETE, 15-HEPE, 5-HEPE, and 14-HDHA) and cyclooxygenase pathway (TXB2). Of note, these metabolites play a critical role in the physiological and pathological functions of inflammation, immunology, and cytokine production. As previously reported by academics, secondary hemophagocytic lymphohistiocytosis (sHLH) is an hyperinflammatory syndrome and often triggered by viral infections, which lead to unremitting fever, cytopenias, increased interleukin (IL)-2, IL-7, granulogytecolony stimulating factor, and interferon-γ–inducible protein 10 in patients with COVID-19 (Huang et al., 2020; Mehta et al., 2020). Moreover, Schwarz et al. (2020) reported that the levels of free PUFAs and cascade metabolites, such as arachidonic acid (AA), eicosapentaenoic acid (EPA), docosapentaenoic acid (DPA), and docosahexaenoic acid (DHA), were enhanced in the COVID-19–infected cohorts, and the changes of these eicosanoids could discriminate the severe from the moderate disease patients. It is known that plasminogen is the main source of PUFAs in immune and structural cells (Lebrero et al., 2019). When systemic immune cells are activated, PUFAs are released from parent glycolipids and subsequently converted to various immune signaling eicosanoids (Dennis and Norris, 2015). The imbalance among pro-inflammatory, immune-regulating, and pro-resolving eicosanoid mediators can not only affect the efficacy of immune response in the process of infectious and sterile inflammatory diseases but also may contribute to disease progression and further change the status of successful treatment of inflammation (Serhan and Savill, 2005). Nowadays, there is no metabolic research regarding remdesivir treatment from this perspective of eicosanoid metabolic disturbance. Our work first revealed the endogenous metabolites trajectories induced by remdesivir treatment, which will benefit in understanding biological pathways related to antiviral drug and initiate mechanistic hypotheses.

It is known that SARS-CoV-2 possibly stimulates cell debris–induced “eicosanoid storm” which leads to a strong inflammatory response in turn (von Moltke et al., 2012). Some eicosanoids, such as resolvins or EETs, could weaken pathological thrombosis and facilitate clot removal, which is becoming a critical pathology of COVID-19 infection (Panigrahy et al., 2020). In this context, metabolites including TXB2, PGE2, and 5-HEPE were all significantly disturbed with VIP > 1 when remdesivir was administered before and after treatment (Figure 4). Conti et al. (2020) reported that IL-1 induces TXB2 releases in activated neutrophils and macrophages, and causes leukocyte aggregation and inflammation, which would explain the dramatic thrombi formation, platelet aggregation, and organ dysfunction in COVID-19. Moreover, results of targeted lipidomic analysis of bronchoalveolar lavages from COVID-19 patients have shown that leukotrienes, and metabolites derived from AA, EPA, and DPA were all increased (Archambault et al., 2021). In the present study, the metabolic levels of DHA, resolvin D2, 5-HEPE, and 5-HETE were decreased after remdesivir treatment (Figure 4), which may provide considerable mirror for understanding the mechanism of COVID-19 and antiviral drugs. Besides, the decrease of resolvin D2 may weaken the anti-inflammatory action to some extent. The reason for this decrease may be attributed to the limited rats in this study. Given that the pivotal role of eicosanoids in SARS-CoV-2 pathogenesis and therapeutic targets, an open-label, randomized, controlled clinical trial (NCT04335032, 2020) was performed in hospitalized patients with confirmed SARS-CoV-2. This clinical trial was classified into two groups: receiving standard care or providing daily 2 g of EPA capsules. The main endpoints comprised the efficacy of EPA, pro-inflammatory IL-6, mortality rate, ICU stays, and mechanical ventilation. Overall, these inflammatory-related metabolites play an important role for the therapy or prevention in patients with COVID-19.

Prostaglandin E2 (PGE2) is the most investigated COX metabolite in modulating innate and adaptive immune cells, and plays a key role in the contact between the two systems, mediated by antigen-presenting cells (APCs) and T lymphocytes (Rogero et al., 2020). Using an animal model and *in vitro* analysis, Coulombe et al. (2014) demonstrated that PGE2 production during influenza A virus (H1N1 strain) infection resulted in the inhibition of type 1 IFN and apoptosis of alveolar macrophages, thereby contributing to increased viral replication. In that case, the inhibition of PGE2 enhanced the antiviral response, indicating that the specific inhibition of PGE2 represents a therapeutic pathway for the cure and prevention of influenza and other potential viral infections. In order to evaluate whether the levels of metabolites correlated with blood drug exposure, correlation analysis between metabolites and pharmacokinetic parameters was conducted and shown in Figure 5. As described, PGE2 correlated with C_max_, AUC_0-t_, AUC_0-infinity_, and Nuc concentrations positively with r > 0.5 and *p* < 0.05. Besides, for the parameter of CL, PGE2 exerts negative correlation. HEPEs which derived from EPA displayed a different tendency. With respect to the correlation analysis between metabolic profile and pharmacokinetics, a PLS model was built after PCA analysis. As shown in Figure 6, a stronger relevance was observed whether for AUC or C_max_, which demonstrated that the most disturbed metabolite (e.g., PGE2) may predict drug response or toxicity.

To date, no available investigation is reported regarding the eicosanoid metabolic fingerprint after remdesivir treatment. The results of the present work will provide metabolomic evidence regarding eicosanoids after treatment of remdesivir *in vivo*. As there have been no effective drug therapy options to treat the SARS-CoV-2 pandemic, although many strengths exist in our study, several limitations had to be mentioned in future works. For instance, a limited number of rats can only provide restricted metabolic data, which may reduce the robustness of the data available in this article. Clinical trials with larger sample sizes will be able to supplement some of the gaps in this study in the future. Second, despite our endeavors to collect blood samples from COVID-19 patients, due to poor sample accessibility, we only examined the metabolomic profiles in healthy rats after a single administration of remdesivir. As reported by Schwarz et al. (2021), compared with the healthy condition, the lipid mediators and eicosanoids were disturbed, including decreased products of ALOX12 (e.g., 12-HEPE, 12-HETE, RvE3, and LXA4) and COX2 (e.g., PGE2, PGD2, PGF2a, TXB2, and 18-HEPE), increase products of ALOX5 (e.g., RvD1-4, LTB4, 5-HEPE, 5-HETE, 7-HDHA, and 7-HDPA), and cytochrome p450 (5,6 DiHETrE, 8,9 DiHETrE, 11,12 DiHETrE, and 14,15 DiHETrE) in human serum. Besides, plasma metabolic results identified 18 metabolites (e.g., LTB4, 9,10-DiHOME, 12,13-DiHOME, PGE2, and PGD2) with statistically significant differences (*p* < 0.01) with more than four-fold change between healthy controls (*n* = 44) and COVID-19 patients (*n* = 6) (McReynolds et al., 2021). Much works, either *in vitro* or *in vivo*, may be necessitated to evaluate metabolomic characteristics under virus attack. Additionally, it will be critical to reproduce novel evidences with an external cohort. Accordingly, external validation datasets should be replenished and verified for better illustrating the robust eicosanoid metabolomics. Taken together, with continuous research and technical advancements, eicosanoid metabolic reprogramming will no doubt provide a notable scientific contribution to the innovation in COVID-19 therapy practice.

## Conclusion

In summary, by means of the robust HPLC-MS/MS targeted method, we first demonstrated eicosanoid metabolic profiles of remdesivir at longitudinal and transversal levels, and correlation with pharmacokinetics. Inherent metabolic phenotype variations of eicosanoids metabolites, such as 15-HETE, 5-HEPE, TXB2, PGE2, and DHA, had turned out after remdesivir treatment. Presently, this study originally provides a systemic and comprehensive eicosanoid metabolomic profiling in rats after intravenous administration of remdesivir, and it also offers strong evidence to the pathology in fighting COVID-19. Collectively, eicosanoid metabolic fingerprint of remdesivir treatment may shed light on therapy development for COVID-19 patients.

## Data Availability

The raw data supporting the conclusion of this article will be made available by the authors, without undue reservation.
