# Supplementary material for: Eicosanoid Metabolomic Profile of Remdesivir Treatment in Rat Plasma by High-Performance Liquid Chromatography Mass Spectrometry
Source: Front Pharmacol. 2021 Sep 29;12:747450. doi: 10.3389/fphar.2021.747450 (PMC8511316; doi:10.3389/fphar.2021.747450)
Supplement: Supplementary file 1 [file DataSheet2.docx]

**Uncertainty of measurement**

As reported in our previous study [1], uncertainty of measurement is an estimate characterizing the range of values within which the true value of the measurand lies. The use of measurement uncertainty depends on two requirements: that a validated method is used for the determination; and an assurance that the material analyzed falls within the scope of the method validation. Standard uncertainty, type A of uncertainty (evaluation by statistical analysis of series of observations), type B uncertainty (evaluation by means other than statistical analysis of series of observations), combined standard uncertainty and expanded uncertainty (*k* = 2, 95% confidence limits) were evaluated according to the guide to the expression of uncertainty in measurement. The procedure for estimation of uncertainty in measurement requires ensuring that analytical process is traceable, establishing performance characteristics of the equipment, and identifying the sources of uncertainty as well as its influence. A serial of Mathematical formula were employed, such as Bessel formula, *S* (*x,M*) = $\sqrt{\frac{\sum_{j=1}^{m} \sum_{k=1}^{n} \left( x_{jk-x_{j}} \right){}^{2}}{m\left( n-1 \right)}}$, *u*_c_ (*m*)=$\sqrt{u^{2}\left( \Delta,{}_{\mathrm{Nonlinear}} \right)+u^{2}(\Delta,{}_{Zeroing})}$, *u* (*x*, *M*)=$\frac{S}{a}\sqrt{\frac{1}{P}+\frac{1}{N}+\frac{{(\bar{x, M}-\bar{x})}^{2}}{S_{xx}}}$, and *u*_c, r,_ *_M_* =$\sqrt{u_{r}^{2}\left( 1, M \right)+u_{r}^{2}\left( 2, M \right)+u_{r}^{2}\left( 3, M \right)+u_{r}^{2}\left( 4, M \right)+u_{r}^{2}\left( 5, M \right)+u_{r}^{2}\left( 6, M \right)+u_{r}^{2}\left( 7, M \right)}$

[1] P. Du, P. Li, R. Zhao, H. Liu, L. Liu, Optimized UPLC-MS/MS method for the quantitation of olanzapine in human plasma: application to a bioequivalence study, Bioanalysis, 11 (2019) 1291-1302
