# Supplementary material for: Eicosanoid Metabolomic Profile of Remdesivir Treatment in Rat Plasma by High-Performance Liquid Chromatography Mass Spectrometry
Source: Front Pharmacol. 2021 Sep 29;12:747450. doi: 10.3389/fphar.2021.747450 (PMC8511316; doi:10.3389/fphar.2021.747450)
Supplement: Supplementary file 2 [file DataSheet1.docx]

**Supplementary Table S1** MRM transition, chromatographic condition, LOD, LLOQ, calibration range and linear regression for eicosanoid metabolites

| Metabolites | CAS NO. | Formula | Precursor ion (*m/z*) | Product ion (*m/z*) | DP | CE | Rention time (min) | LOD  (S/N=3) (ng mL^-1)^ | LLOQ  (S/N=5) (ng mL^-1)^ | linearity (ng mL^-1)^ | Correlation (*r*) |
| --- | --- | --- | --- | --- | --- | --- | --- | --- | --- | --- | --- |
| 17(18)-EpETE | / | C20H30O3 | 317 | 259,299,255,215 | -50 | -16 | 8.21 | 0.02 | 0.05 | 0.05-500 | 0.997 |
| 19(20)-EpDPA | / | C22H32O3 | 343 | 299,281,241,325 | -50 | -17 | 10.1 | 0.2 | 0.5 | 0.5-500 | 0.99812 |
| 9-HEPE | 286390-03-2 | C20H30O3 | 317 | 167,255,273,149 | -70 | -25 | 7.41 | 0.05 | 0.1 | 0.1-20 | 0.99818 |
| 15-HETE | 71030-36-9 | C20H32O3 | 319 | 219,175,257,301 | -20 | -20 | 8.14 | 0.02 | 0.05 | 0.05-500 | 0.99855 |
| 16-HETE | 128914-46-5 | C20H32O3 | 319 | 233,257,189,301 | -25 | -20 | 7.32 | 0.02 | 0.05 | 0.05-500 | 0.99777 |
| PGB3 | 36614-32-1 | C20H28O4 | 331 | 202,175,262,313 | -40 | -26 | 3.68 | 0.02 | 0.05 | 0.05-500 | 0.99615 |
| 12-HEPE | 81187-21-5 | C20H30O3 | 317 | 179,255,135,299 | -40 | -20 | 7.2 | 0.02 | 0.05 | 0.05-500 | 0.99862 |
| 5-Oxo-EE | 106154-18-1 | C20H30O3 | 317 | 203,245,273,299 | -90 | -24 | 11.14 | 0.05 | 0.1 | 0.1-500 | 0.99876 |
| 5(6)-DiHET | 213382-49-1 | C20H34O4 | 337 | 145,191,255,319 | -60 | -23 | 7.05 | 0.05 | 0.1 | 0.1-500 | 0.99722 |
| 8(9)-EpETE | 851378-93-3 | C20H30O3 | 317 | 255,203,273,299 | -65 | -16 | 9.19 | 0.1 | 0.1 | 0.2-500 | 0.99854 |
| 14-HDHA | 87042-40-8 | C22H32O3 | 343 | 205,281,161,234 | -55 | -18 | 8.72 | 0.05 | 0.1 | 0.1-500 | 0.99807 |
| 9-HETE | 79495-85-5 | C20H32O3 | 319 | 167,257,179,301 | -46 | -20 | 9.33 | 0.05 | 0.1 | 0.1-500 | 0.99883 |
| 11-HEPE | 99217-78-4 | C20H30O3 | 317 | 167,195,255,299 | -50 | -20 | 6.95 | 0.05 | 0.05 | 0.1-500 | 0.99685 |
| 5-HETE | 73307-52-5 | C20H32O3 | 319 | 115,203,257,301 | -63 | -20 | 9.76 | 0.02 | 0.01 | 0.05-500 | 0.99886 |
| 17-HETE | 128914-47-6 | C20H32O3 | 319 | 247,203,257,301 | -70 | -20 | 7.23 | 0.05 | 0.05 | 0.1-500 | 0.99797 |
| LTE4 | 75715-89-8 | C23H37NO5S | 438 | 333,351,420,235 | -30 | -26 | 3.94 | 0.05 | 0.1 | 0.1-500 | 0.99807 |
| LTD4 | 73836-78-9 | C25H40N2O6S | 495 | 177,143,477 | -52 | -26 | 3.71 | 0.02 | 0.05 | 0.05-500 | 0.99831 |
| 14(15)-EpEDE | 351533-80-7 | C20H34O3 | 321 | 221,113,209,303 | -83 | -19 | 11.95 | 0.05 | 0.1 | 0.1-500 | 0.99785 |
| 11,12-EpETE | 504435-15-8 | C20H30O3 | 317 | 167,179,255,299 | -25 | -19 | 8.98 | 0.02 | 0.05 | 0.05-500 | 0.99707 |
| 16(17)-EpDPA | 155073-46-4 | C22H32O3 | 343 | 233,299,281,259 | -35 | -16 | 10.75 | 0.1 | 0.2 | 0.2-500 | 0.9987 |
| 20-HDHA | 90906-41-5 | C22H32O3 | 343 | 281,241,133,299,325 | -52 | -19 | 10.75 | 0.5 | 0.5 | 0.5-500 | 0.99736 |
| 11-HDHA | 87018-59-5 | C22H32O3 | 343 | 121,149,281,165,325 | -35 | -20 | 9.06 | 0.02 | 0.05 | 0.05-500 | 0.99864 |
| 8-HDHA | 90780-54-4 | C22H32O3 | 343 | 189,109,135 | -70 | -16 | 9.46 | 0.02 | 0.05 | 0.05-500 | 0.9978 |
| 20-hydroxy LTB4 | 79516-82-8 | C20H32O5 | 351 | 195,333 | -52 | -24 | 2.7 | 0.02 | 0.05 | 0.05-200 | 0.99789 |
| PGG2 | 51982-36-6 | C20H32O6 | 367 | 235,187,269 | -74 | -13 | 3.5 | 0.02 | 0.05 | 0.05-500 | 0.99305 |
| LTC4 | 72025-60-6 | C30H47N3O9S | 624 | 272,254,306,606 | -53 | -32 | 3.78 | 0.05 | 0.1 | 0.1-500 | 0.99689 |
| 5(S)-HpETE | 71774-08-8 | C20H32O4 | 335 | 203,317,129,273 | -82 | -30 | 4.84 | 0.2 | 0.5 | 0.5-500 | 0.9979 |
| LTB3 | 88099-35-8 | C20H34O4 | 337 | 195,122,151,319 | -82 | -22 | 5.84 | 0.02 | 0.05 | 0.05-500 | 0.99917 |
| 20-HETE | 79551-86-3 | C20H32O3 | 319 | 289,275,257,301 | -48 | -28 | 6.75 | 0.02 | 0.02 | 0.02-500 | 0.99728 |
| 5(S)-HpEPE | 143292-98-2 | C20H30O4 | 333 | 155,201,173,271 | -28 | -16 | 8.21 | 0.5 | 1 | 1-500 | 0.99792 |
| 12-HETE | 71030-37-0 | C20H32O3 | 319 | 179,257,135,301 | -40 | -22 | 11.18 | 0.1 | 0.2 | 0.2-500 | 0.99701 |
| 5-HEPE | 83952-40-3 | C20H30O3 | 317 | 115,255,299,201 | -50 | -17 | 7.75 | 0.01 | 0.02 | 0.02-500 | 0.99855 |
| 11(12)-EET | 123931-40-8 | C20H32O3 | 319 | 167,179,257,301 | -55 | -19 | 11.18 | 0.05 | 0.05 | 0.05-500 | 0.99725 |
| 18-HEPE | 141110-17-0 | C20H30O3 | 317 | 259,255,299,273,215 | -49 | -19 | 6.4 | 0.05 | 0.05 | 0.05-500 | 0.99741 |
| LTF4 | 83851-42-7 | C28H44N2O8S | 567 | 171,127,351,438 | -47 | -30 | 3.91 | 0.1 | 0.1 | 0.1-500 | 0.99684 |
| 15-Oxo-ETE | 81416-72-0 | C20H30O3 | 317 | 113,273,139,299 | -34 | -22 | 8.71 | 0.05 | 0.05 | 0.05-500 | 0.99889 |
| LTB4 | 71160-24-2 | C20H32O4 | 335 | 195,273,151,317 | -29 | -20 | 4.84 | 0.02 | 0.05 | 0.05-500 | 0.99795 |
| 11,12-DiHETrE | / | C20H34O4 | 337 | 167,257,319 | -49 | -24 | 5.94 | 0.01 | 0.02 | 0.02-500 | 0.99885 |
| 5(6)-DiHETE | 845673-97-4 | C20H32O4 | 335 | 145,273,205,317 | -64 | -24 | 5.58 | 1 | 1 | 1-500 | 0.99762 |
| 14(15)-DiHET | / | C20H34O4 | 337 | 207,129,163,319 | -40 | -24 | 5.51 | 0.02 | 0.02 | 0.02-500 | 0.99795 |
| 15-HEPE | 88852-33-9 | C20H30O3 | 317 | 219,255,175,299 | -75 | -18 | 6.83 | 0.01 | 0.02 | 0.02-500 | 0.99744 |
| 8(9)-EET | / | C20H32O3 | 319 | 155,257,167,275 | -44 | -17 | 11.51 | 0.02 | 0.05 | 0.05-500 | 0.99715 |
| 19-HETE | 115461-39-7 | C20H32O3 | 319 | 275,231,257,301 | -50 | -23 | 6.63 | 0.1 | 0.2 | 0.2-500 | 0.99613 |
| 13-HDHA | 90780-53-3 | C22H32O3 | 343 | 193,281,221,325 | -30 | -16 | 8.53 | 0.05 | 0.1 | 0.1-500 | 0.99415 |
| 5(6)-EET | 87173-80-6 | C20H32O3 | 319 | 191,275,257,301 | -35 | -16 | 11.89 | 0.05 | 0.1 | 0.1-500 | 0.99806 |
| Resolvin D1 | 872993-05-0 | C22H32O5 | 375 | 141,215,233,277 | -32 | -22 | 3.4 | 0.02 | 0.02 | 0.02-500 | 0.99688 |
| Resolvin D2 | 810668-37-2 | C22H32O5 | 375 | 175,141,215,277 | -30 | -30 | 3.61 | 0.05 | 0.1 | 0.1-500 | 0.99573 |
| PGE3 | 802-31-3 | C20H30O5 | 349 | 331,313,269,233 | -34 | -13 | 3.06 | 0.05 | 0.1 | 0.1-500 | 0.99516 |
| 11-dehydro TXB2 | 67910-12-7 | C20H32O6 | 367 | 305,349,243,161 | -69 | -21 | 3.33 | 0.05 | 0.1 | 0.1-500 | 0.99781 |
| PGJ2 | 60203-57-8 | C20H30O4 | 333 | 271,189,233,315 | -34 | -19 | 4.04 | 0.05 | 0.1 | 0.1-500 | 0.99731 |
| TXB1 | 64626-32-0 | C20H36O6 | 371 | 171,197,327 | -59 | -22 | 3.08 | 0.02 | 0.05 | 0.05-500 | 0.9955 |
| PGD2 | 41598-07-6 | C20H32O5 | 351 | 271,189,315,233 | -61 | -15 | 3.41 | 0.1 | 0.2 | 0.2-500 | 0.99568 |
| PGI2 | 61849-14-7 | C20H31O5 | 351 | 315,271,189,233 | -53 | -21 | 3.3 | 0.1 | 0.2 | 0.2-500 | 0.99157 |
| 15-deoxy-Δ12,14-PGJ2 | 87893-55-8 | C20H28O3 | 315 | 271,203,297 | -35 | -23 | 6.66 | 0.02 | 0.05 | 0.05-500 | 0.99709 |
| 6-keto PGF1α | 58962-34-8 | C20H34O6 | 369 | 163,245,207,315 | -25 | -35 | 2.6 | 0.01 | 0.02 | 0.02-500 | 0.99678 |
| 8(9)-DiHET | / | C20H34O4 | 337 | 127,185,257,319 | -65 | -28 | 6.38 | 0.005 | 0.005 | 0.005-500 | 0.99684 |
| 17-HDHA | 90780-52-2 | C22H32O3 | 343 | 201,281,245,325 | -33 | -19 | 8.34 | 0.05 | 0.1 | 0.1-500 | 0.99362 |
| DHA | 6217-54-5 | C22H32O2 | 327 | 283,299,249,309 | -34 | -15 | 16.08 | 0.05 | 0.1 | 0.1-500 | 0.9979 |
| Lipoxin A4 | 89663-86-5 | C20H32O5 | 351 | 115,217,235,333 | -57 | -21 | 3.62 | 0.01 | 0.02 | 0.02-500 | 0.99713 |
| Arachidonic Acid | 506-32-1 | C20H32O2 | 303 | 259,205 | -71 | -30 | 16.25 | 0.01 | 0.02 | 0.02-100 | 0.99572 |
| Maresin-1 | 1268720-28-0 | C22H32O4 | 359 | 177,250,297,341 | -60 | -21 | 4.64 | 0.05 | 0.1 | 0.1-500 | 0.99889 |
| 20-carboxy LTB4 | 80434-82-8 | C20H30O6 | 365 | 347,169,303,195 | -39 | -24 | 2.99 | 0.2 | 0.5 | 0.5-200 | 0.99551 |
| LTB5 | 80445-66-5 | C20H30O4 | 333 | 195,129,315 | -19 | -23 | 4.12 | 0.02 | 0.05 | 0.05-200 | 0.99702 |
| 6-keto PGE1 | 67786-53-2 | C20H32O6 | 367 | 143,331,205,349 | -25 | -26 | 2.74 | 0.05 | 0.1 | 0.1-500 | 0.99594 |
| TXB2 | 54397-85-2 | C20H34O6 | 369 | 169,195,177,325 | -24 | -22 | 3.16 | 0.05 | 0.1 | 0.1-500 | 0.9977 |
| PGE2 | 363-24-6 | C20H32O5 | 351 | 271,189,315,233 | -54 | -21 | 3.41 | 0.02 | 0.05 | 0.05-500 | 0.99573 |
| PGB2 | 13367-85-6 | C20H30O4 | 333 | 175,235,271,315 | -52 | -28 | 4.11 | 0.1 | 0.2 | 0.2-500 | 0.99815 |
| TXB3 | 71953-80-5 | C20H32O6 | 367 | 169,195,177,125 | -67 | -21 | 3 | 0.05 | 0.1 | 0.1-500 | 0.99645 |

**Supplementary Table S2.** Intra- and inter-day accuracy and precision of HPLC-MS/MS method for determining Nuc in rat plasma (*n*=6)

| Conc. (ng/mL) | Intra-day | | |  | Inter-day | | |
| --- | --- | --- | --- | --- | --- | --- | --- |
|  | Mean±SD | CV (%) | Bias (%) |  | Mean±SD | CV (%) | Bias (%) |
| 2 | 1.99±0.19 | 9.55 | -0.50 |  | 1.94±0.17 | 8.97 | -3.09 |
| 5 | 5.02±0.3 | 5.98 | 0.40 |  | 5.15±0.41 | 7.99 | 3.06 |
| 10 | 10.12±0.83 | 8.20 | 1.20 |  | 9.96±0.87 | 8.76 | -0.44 |
| 100 | 100.25±4.43 | 4.42 | 0.25 |  | 101.82±7.64 | 7.50 | 1.82 |
| 800 | 792.5±52.56 | 6.63 | -0.94 |  | 760.13±56.78 | 7.47 | -4.98 |
