# Supplementary figures and images for: Eicosanoid Metabolomic Profile of Remdesivir Treatment in Rat Plasma by High-Performance Liquid Chromatography Mass Spectrometry
Source: Front Pharmacol. 2021 Sep 29;12:747450. doi: 10.3389/fphar.2021.747450 (PMC8511316; doi:10.3389/fphar.2021.747450)

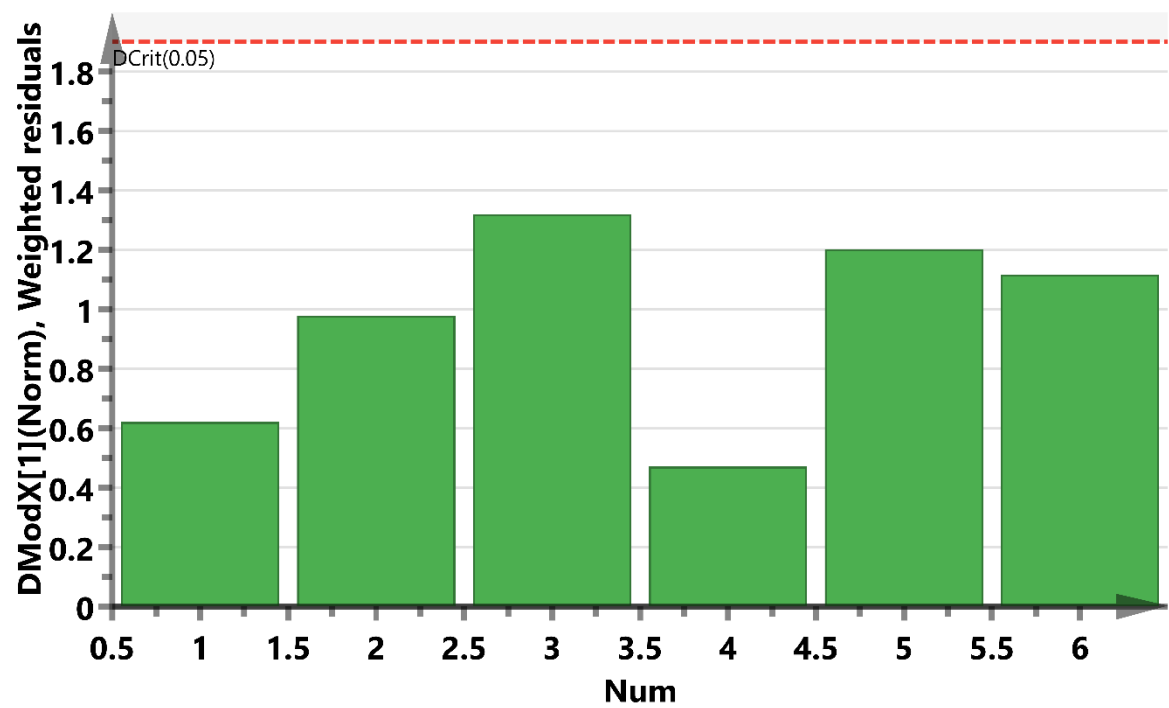

Supplementary Figure S1. The distance to the model plot for identifying the outliers.

Supplement: Supplementary file 3 [file Image1.pdf]
